# Martian dunes indicative of wind regime shift in line with end of ice age

**DOI:** 10.1038/s41586-023-06206-1

**Published:** 2023-07-05

**Authors:** Jianjun Liu, Xiaoguang Qin, Xin Ren, Xu Wang, Yong Sun, Xingguo Zeng, Haibin Wu, Zhaopeng Chen, Wangli Chen, Yuan Chen, Cheng Wang, Zezhou Sun, Rongqiao Zhang, Ziyuan Ouyang, Zhengtang Guo, James W. Head, Chunlai Li

**Affiliations:** 1 Key Laboratory of Lunar and Deep Space Exploration, National Astronomical Observatories, Chinese Academy of Sciences, Beijing, China; 2 Key Laboratory of Cenozoic Geology and Environment, Institute of Geology and Geophysics, Chinese Academy of Sciences, Beijing, China; 3 State Key Laboratory of Tibetan Plateau Earth System, Resources and Environment, Institute of Tibetan Plateau Research, Chinese Academy of Sciences, Beijing, China; 4 Beijing Aerospace Control Center, Beijing, China; 5 Beijing Institute of Spacecraft System Engineering, Beijing, China; 6 Lunar Exploration and Space Engineering Center, Beijing, China; 7 Institute of Geochemistry, Chinese Academy of Sciences, Guiyang, China; 8 Department of Earth, Environmental and Planetary Sciences, Brown University, Providence, RI USA

**Keywords:** Planetary science, Planetary science

## Abstract

Orbital observations suggest that Mars underwent a recent ‘ice age’ (roughly 0.4–2.1 million years ago), during which a latitude-dependent ice-dust mantle (LDM)^1,2^ was emplaced. A subsequent decrease in obliquity amplitude resulted in the emergence of an ‘interglacial period’^1,3^ during which the lowermost latitude LDM ice^4–6^ was etched and removed, returning it to the polar cap. These observations are consistent with polar cap stratigraphy^1,7^, but lower- to mid-latitude in situ surface observations in support of a glacial–interglacial transition that can be reconciled with mesoscale and global atmospheric circulation models^8^ is lacking. Here we present a suite of measurements obtained by the Zhurong rover during its traverse across the southern LDM region in Utopia Planitia, Mars. We find evidence for a stratigraphic sequence involving initial barchan dune formation, indicative of north-easterly winds, cementation of dune sediments, followed by their erosion by north-westerly winds, eroding the barchan dunes and producing distinctive longitudinal dunes, with the transition in wind regime consistent with the end of the ice age. The results are compatible with the Martian polar stratigraphic record and will help improve our understanding of the ancient climate history of Mars^9^.

## Abstract (web summary)

Evidence for a stratigraphic sequence involving initial barchan dune formation, with the transition in wind regime consistent with the end of the ice age is found, compatible with the Martian polar stratigraphic record.

## Main

**Fig. 1:**
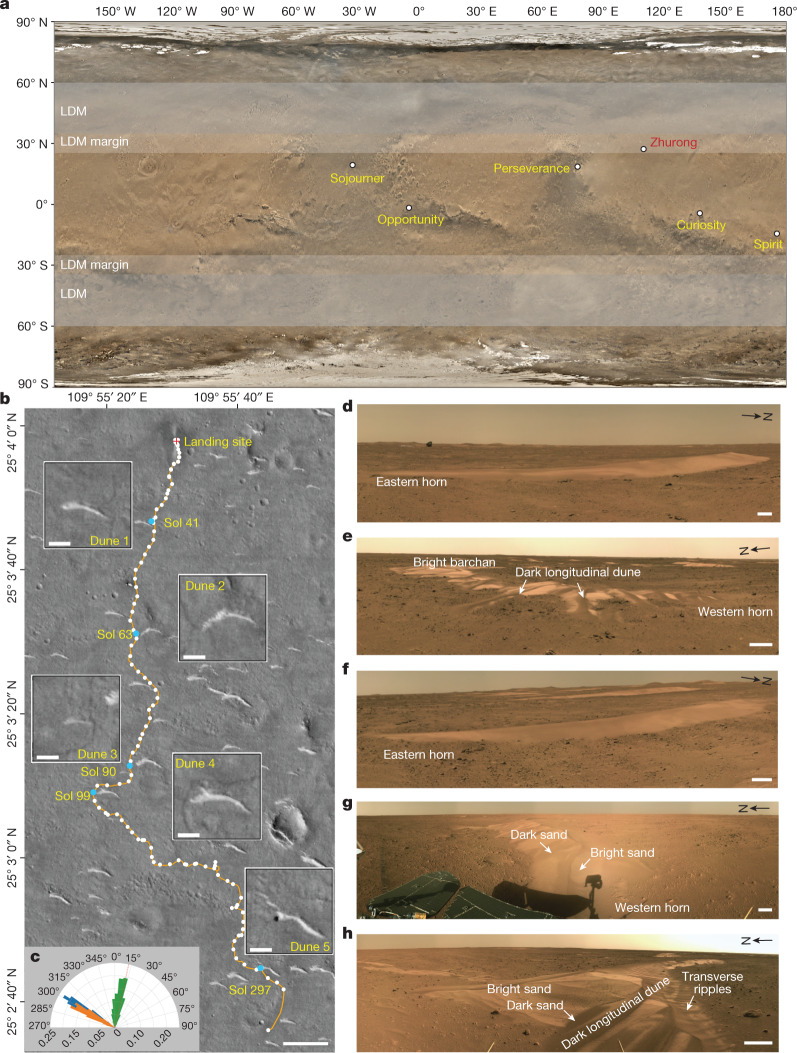
Mars rovers landing site and dune investigations in the Zhurong exploration zone. **a**, The landing site of previous and continuing Mars rover-scale exploration. The base map shows the global remote sensing images of Moderate Resolution Camera onboard Tianwen-1. **b**, The traverse map of the Zhurong rover. The base image shows HiRIC remote sensing images of the Zhurong landing zone on 16 March 2022. The blue line marks the field of view of the dunes image in **d**–**h** . Scale bars in boxes, 10 m; scale bar, 100 m. **c**, The inferred wind directions from the bright barchans and the landforms on their western and eastern horns. Green represents the wind direction indicated by the bright barchans, orange represents the wind direction indicated by the thin tail on the east horns of the bright dunes, and blue represents the wind direction indicated by the dark longitudinal dunes on the west horns of the bright barchans. **d**–**h**, Panoramic images of the dunes taken by the NaTeCam on the Zhurong rover: dune 1 (**d**), dune 2 (**e**), dune 3 (**f**), dune 4 (**g**) and dune 5 (**h**). Scale bars, 0.5 m. Credit: CNSA/GRAS.

Conclusive evidence for the nature and causes of Mars climate history has been elusive due to the extreme complexity of past weather, climate and atmosphere, and the spin-axis or orbital parameters known to exert strong climate influence^3,10^. Forward-modelling, from the ancient geological record to today, has been used, but key missing elements of the early geological record have precluded a robust pathway. Some have used inverse modelling, using known current conditions and the most recent geological record (polar deposits and the stratigraphically youngest geologic units) to infer recent climate conditions, an approach made more powerful by the robust prediction of spin-axis or orbital conditions for the last 20 Myr (million years)^3^. The inverse modelling approach has provided orbital geological evidence for a recent ice age^1^ during which increased obliquity amplitude mobilized polar ice and deposited a metres-thick ice and dust layered mantle down to the lower mid-latitudes; this decreased the obliquity amplitude roughly 0.4 Ma (million years ago)^3^ and mobilized marginal LDM ice, returning it to the polar layered terrain^1^. To test this hypothesis, detailed rover-scale observations of feature morphology and stratigraphy are needed at the most active southern LDM margin^1,2,4,5^ that can be linked to atmospheric mesoscale and global climate models and the predicted climate history^3,10^. As shown during previous rover-scale exploration (Methods), aeolian landforms, typically the youngest features in the stratigraphic column, provide critical information on recent climate-related processes and can also provide links to wind directions, surface alteration environments and aeolian processes in general. This previous and continuing rover-scale exploration has been confined to low-latitude sites (less than 20º from the equator) (Fig. 1a), lying below the latitudes of the areas predicted to be the most dynamic (the southern LDM margin at 25–35º latitude). China’s Tianwen-1 or Zhurong rover was targeted to land in this critical LDM transition region (25.066° N), known from orbital data to have abundant aeolian features, and has traversed 1,921 m in a southerly direction normal to the LDM margin (Fig. 1a,b), making critical in situ observations of aeolian features, data essential to provide links to atmospheric model predictions^1,3,10,11^. The Zhurong scientific payload (Methods) is ideally suited to provide data to test recent climate change hypotheses^1,3,10^. Here we describe observations of the dunes in the region using both Zhurong rover and orbital data, derive the nature of the wind fields and their changing patters as interpreted from stratigraphic relationships, assess the relation to global climate models^10^ and recent obliquity changes^1,11^, and synthesize the data into an interpretation of the geologically very recent climate history of Mars.

## Zhurong landing zone dune observations

Among recent Martian aeolian features, bright decametre-width transverse aeolian ridges (TARs) have simple morphologies, occur mainly at medium-low latitudes and their crests are transverse to local winds^12–17^; their nature and formation mechanisms have been studied from orbit^12^ for decades. TAR ages, compositions, factors controlling their distribution and their roles in the global sediment cycle are poorly understood; surface exploration has progressed^17–19^ but has been hindered by lack of in situ information on current meteorology (wind direction, intensity) and concurrent information on current and recent alteration environments (surface temperature variations, humidity, surface alteration geochemistry) and their stratigraphic relationships. General circulation models (GCM) have difficulties reconciling TAR-predicted wind fields, highlighting the need for improved rover-scale knowledge of the boundary conditions necessary to improve GCM models^20,21^.

**Extended Data Fig. 1:**
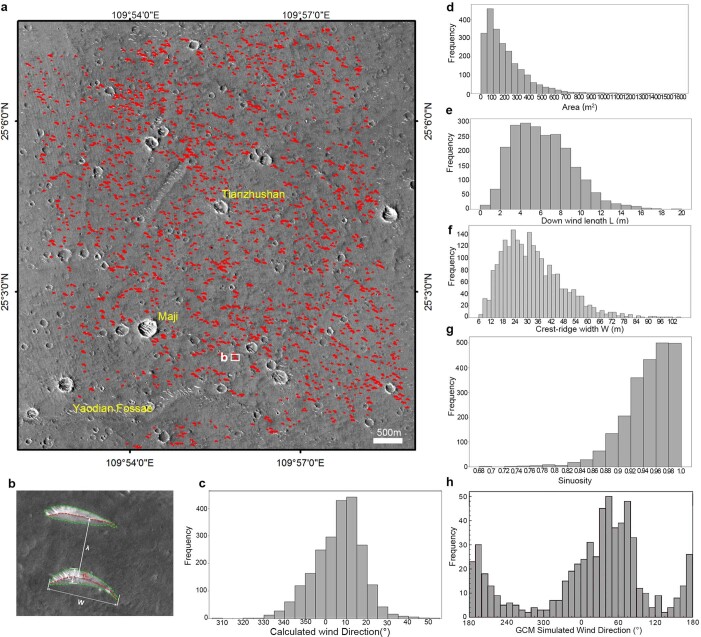
Morphologic statistics of bright barchans around the Zhurong landing site. **a**, the distribution of 2262 bright barchans extracted from HiRIC image, marked by red lines. **b**, Example of morphometric parameters used to describe the bright barchans. Their morphological parameters include area (region enclosed by green dashed line), the crest-ridge width (*W*), the “down wind” length (*L*), crestline length (r*L*) (denoted by red dashed line) and crest to crest wavelength (*λ*). **c**, Frequency distributions of wind direction calculated by referring to crestline orientation. **d**–**g**, Frequency distributions of area, downwind length (*L*), the crest-ridge width (*W*), and sinuosity(*W*/r*L*) of the dunes. **h**, Frequency distributions of wind direction simulated by GCM with the average solar scenario at the Zhurong landing site. The simulation time range is Mars Years 24 to 31.

The Zhurong exploration zone is relatively flat (average slope roughly 2.5°) and shows numerous bright barchans with decametre dune widths (Fig. 1b). Compared with ‘saturated’ or ‘closely spaced’ TARs, their distribution is more widely spaced, unconnected and uniform (Methods and Extended Data Fig. 1). These isolated bright barchans, developed on flat regions, all have generally the same orientation and are not influenced by local topography, supporting the interpretation that the aeolian processes are controlled by the regional climatic environment.

Five dunes were investigated on the rover traverse (Fig. 1b,d–h and Supplementary Table 2). Each of the bright dunes is an eroded barchan. Bright sands form the main barchan body and dark sands clearly overlay the bright dunes. The dark sand accumulations are characterized by small longitudinal dunes, transverse ripples and ridges of surficially coated sands; their colour is similar to nearby soils (Fig. 1d–h).

**Fig. 2:**
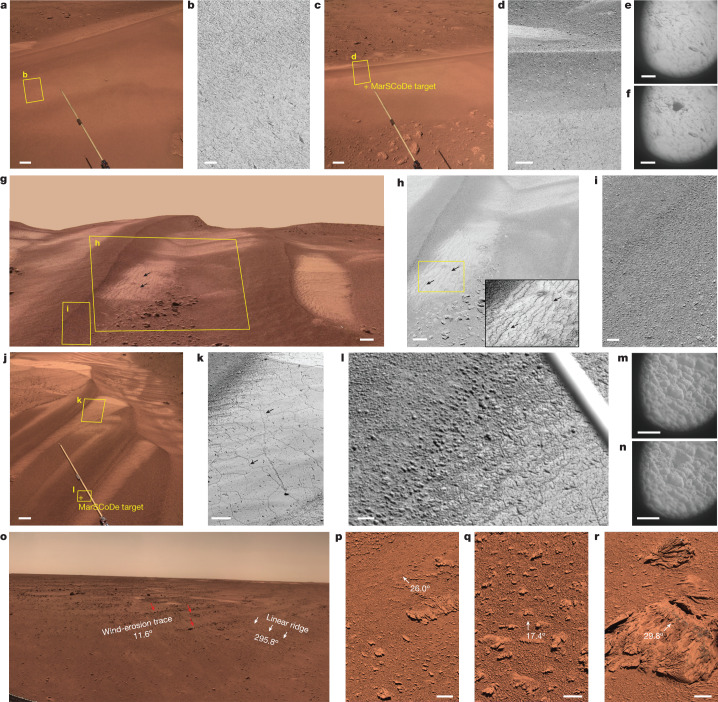
Morphological features of bright sands, dark sands, wind-erosion traces, windblown linear ridges and ventifacts in the Zhurong landing zone. **a**,**b**, NaTeCam image (**a**) and MSCam image (**b**) (525 nm, 768 × 512 pixels) of the bright sand on dune 1. Scale bars, 10 cm (**a**) and 2 cm (**b**). **c**,**d**, NaTeCam image and MSCam image (525 nm, 768 × 512 pixels) of the sand ridge and bright sand on dune 3. Scale bars, 10 cm (**c**) and 2 cm (**d**). **e**,**f**, MarSCoDe micro-images of the bright sand surfaces on dune 3 before (**e**) and after (**f**) being hit by LIBS laser shots. The position of laser shots is shown in in the crosshair of **c**. Scale bars, 5 mm. **g**, A partial 3D scene for dune 2 derived from NaTeCam stereo images. Scale bar, 10 cm. **h**, MSCam image (525 nm, 2,048 × 2,048 pixels) of parallel cracks on the surface of bright barchans (dune 2). Scale bar, 6 cm. **i**, MSCam image (525 nm, 768 × 512 pixels) of the dark sands on dune 2. Scale bar, 2 cm. **j**,**k**, NaTeCam image (**j**) and MSCam image (**k**) (525 nm, 768 × 512 pixels) of polygonal cracks on the surface of bright barchans (dune 5). Scale bars, 20 cm (**j**) and 5 cm (**k**). **l**, MSCam image (525 nm, 512 × 768 pixels) of the dark sands on dune 5. Scale bar, 2 cm. **m**,**n**, MarSCoDe micro-images of the dark sands surfaces on dune 5 before (**m**) and after (**n**) being hit using LIBS laser shots. The position of laser shots on Dune 5 is shown in the crosshair of **j**. Scale bars, 5 mm. **o**, The wind-erosion trace and windblown linear ridges indicated two different wind directions from the north-east and north-west, respectively, in NaTeCam images. The images were taken on Sol 41. **p**–**r**, Ventifacts with grooves and etchings on the windward sides or along wind direction in MSCam colour images (768 × 512 pixels), which were taken on Sol 100 (**p**), Sol 69 (**q**) and Sol 79 (**r**). Scale bars, 5 cm. Credit: CNSA/GRAS.

Bright barchan orientations suggest that they formed from a wind direction transverse to the crest; thus the predominant wind direction was from the north-east (azimuth of 12.5°) (Fig. 1c and Extended Data Fig. 1c). Zhurong high-resolution images reveal that the bright barchan surfaces are encrusted and relatively smooth (Fig. 2a–f). Bright sand surfaces show abundant cracks, polygon-shaped or parallel to topographic contour lines (Fig. 2g,h,j,k). Such small centimetre-scale cracks have been observed on dunes before at Gale Crater^17^, and are generally interpreted to be associated with high desiccation rates and thin stressed regions, conditions commonly associated with surface evaporation^22^.

**Extended Data Fig. 2:**
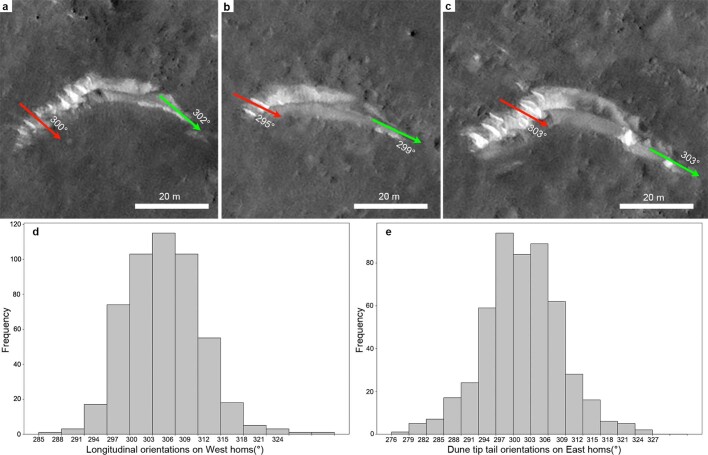
The morphologic features and metrics of bright barchans modified by NW wind. **a**–**c**, Three typical modified dunes identified from HiRISE images. Red arrows indicate the orientations of longitudinal dunes on their western horns, and green arrows indicate the orientations of tip tails on their eastern horns. **d**,**e**, are frequency distributions of the orientations for longitudinal dunes and tip tails of 500 significantly modified dunes, respectively.

**Extended Data Fig. 3:**
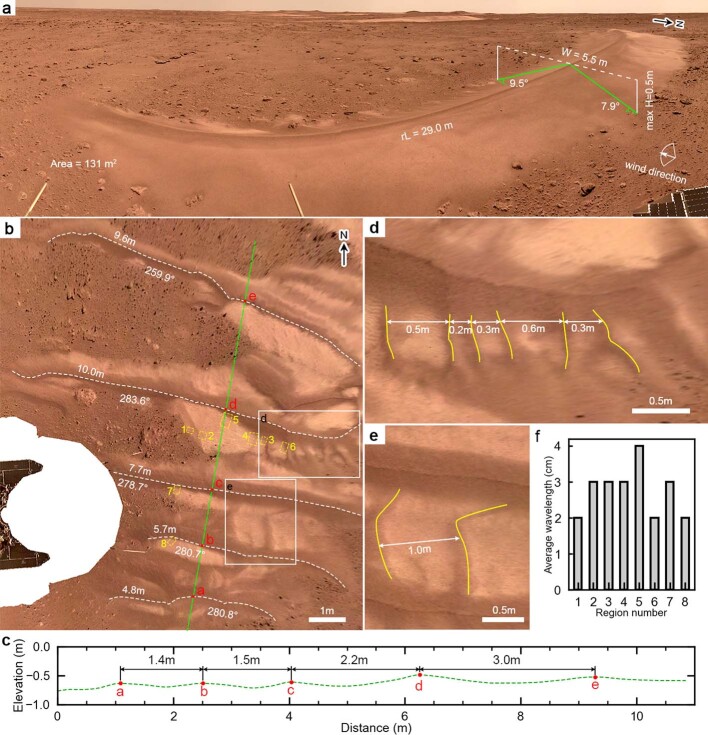
The definition of morphological metrics and their measurements on Dune 3 and longitudinal dunes of Dune 2. **a**, Morphological metrics of Dune 3. **b**, DOM of longitudinal dunes on the western horns of Dune 2. The white dashed line shows the five identified longitudinal dunes. **c**, the topographic profile (green dashed line) perpendicular to the orientation of longitudinal dunes; **d**,**e**, The measurement results of spacings among the transverse ripples in the corresponding rectangular region in **b**. **f**, Statistical results for the wavelength of the parallel cracks measured at 8 regions (dashed-lined yellow boxes of No. 1–8 in **b**).

**Extended Data Fig. 4:**
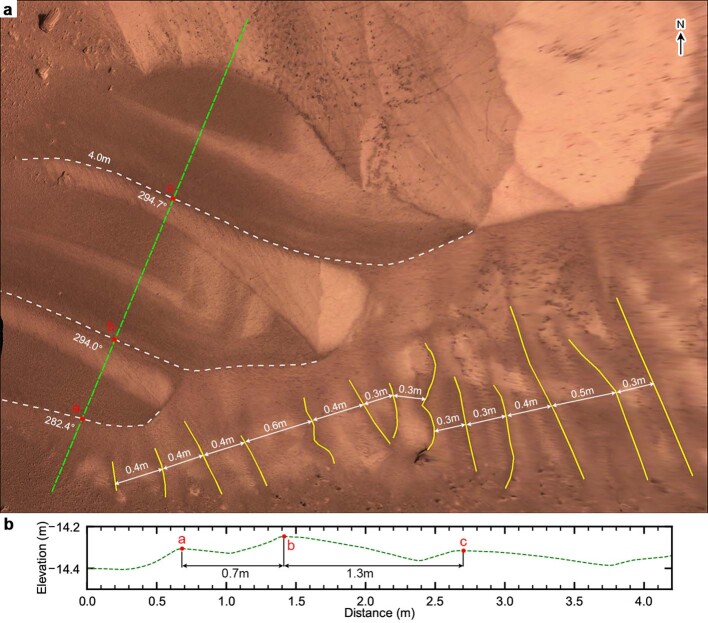
The definition of morphological metrics and their measurements on the longitudinal dunes of Dune 5. **a**, DOM of the west horns of Dune 5, where 3 longitudinal dunes in white dashed line and 13 transverse ripples in yellow line were noted. **b**, the elevation profile following the green dashed line in a, perpendicular to the longitudinal dunes.

Notably, the five barchans are modified to an extent and partly eroded. The dunes show an asymmetrical east to west shape. Eastern barchan horns are sharp and narrow; by contrast, western horns are short and wide, showing north-west-oriented dark longitudinal dunes (Fig. 1d–h). The dark longitudinal dunes of dunes 2 and 5 western horns are the most prominent (Fig. 1e,h), with mean azimuths oriented from 283.9° and 290.4°, respectively (Methods, Extended Data Figs. 3b and 4a and Supplementary Table 1). This is consistent with orientations measured from orbital images (Fig. 1c, Extended Data Fig. 2 and Supplementary Table 1). These relationships demonstrate that bright barchan horns were modified postemplacement and then reformed under a northwestern wind field (azimuth roughly 300°). Longitudinal dunes are much smaller than large dark dunes in other regions^23^ (up to several tens of metres), potentially indicating inadequate sand sources. Close inspection of dunes 2 and 5 longitudinal dunes shows that the north and south slopes are not symmetrical and their orientations differ (Extended Data Figs. 3b and 4a), suggesting variable wind fields.

**Extended Data Fig. 5:**
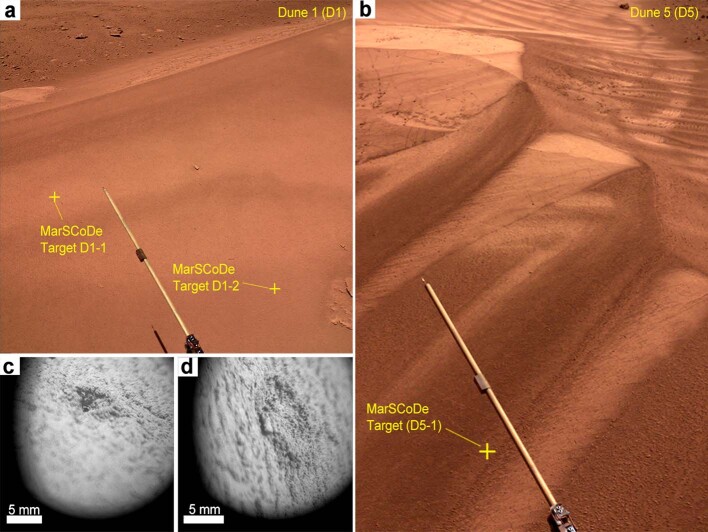
The observations of MarSCoDe on Dune 1 and 5. **a**,**b**,The NaTeCam images of Dune 1 and 5, where yellow crosses denote the target positions of MarSCoDe. **c**,**d**, The MarSCoDe micro-images of the bright and dark sands surfaces on Dune 1 after being hit using LIBS laser shots.

Abundant coarse grains (3–15 mm diameter) were found on longitudinal dunes, sand ridges and surficial-coated sands (Fig. 2d,i,l–n). These particle grain-sizes fall far from the size-range of saltating grains^24^. In response to laser shots (laser-induced breakdown spectrometer, LIBS) (Fig. 2m,n and Extended Data Fig. 5c,d) these grains turned into fine powders (100–300 µm) similar to those of the bright barchan surfaces, strongly suggesting that the coarser grains are agglomerated particles cemented from smaller sand grains.

In addition, Zhurong images clearly show wind-erosion traces, linear ridges and ventifacts, and their orientations reflect two different wind directions from the north-east (azimuth 11.6°–29.8°) and the north-west (azimuth 295.8°–292.7°) (Fig. 2o–r and Supplementary Table 1). These orientations are in accordance with the predominant wind directions inferred from bright barchans and dark longitudinal dunes, suggesting that surficial soil erosion may be providing grains for dune deposition during the two regimes.

Zhurong Mars Surface Composition Detection Package (MarSCoDe) LIBS spectra revealed that the bright and dark sands have chemical compositions similar to average Martian soils, close to basaltic (Supplementary Table 3). Bright sands contain slightly more Mg than dark sands. Short-wave infrared (SWIR) spectra reveal dunes absorption features associated with hydrated sulfates and chlorides (Methods and Extended Data Fig. 7). MSCam images revealed apparent differences in surface roughness between bright barchans and dark dunes: dark dune roughness parameters are nearly twice as high as bright barchans (Methods). In addition, there may be more fine dust grains in the cemented crusts of bright barchans^25^ and experiments showed that only roughly 3 × 10^−4^ g cm^−^^2^ of dust can significantly raise the albedo of darker Martian surfaces^26^. These two factors are the most likely explanation for bright-dark sediment albedo differences.

## Paleoclimate inferred from dunes

**Fig. 3:**
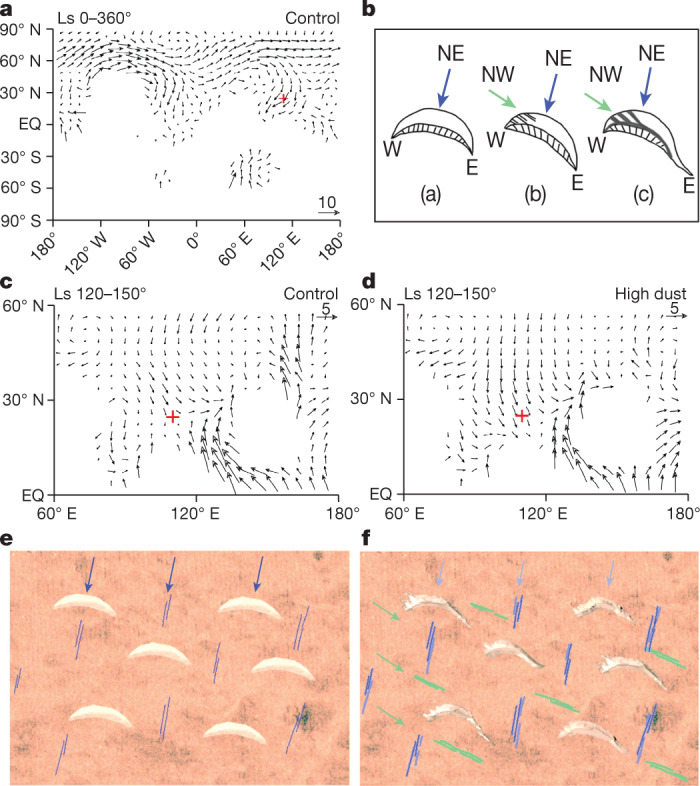
The wind field and the formation model of dunes in the Zhurong landing zone. **a**, Simulated annual mean surface wind field on modern Mars (obliquity 25.19°) by the control parameters of the LMD.Mars (Le Laboratoire de Météorologie Dynamique, Martian Atmospheric GCM) under the climatological condition (that is, control experiment)^35,36^. **c**,**d**, The surface wind field during *L*_s_ = 120–150° on modern Mars by the control parameters (**c**) and high dust (**d**) experiment with prescribed high dust scenario of LMD.Mars. The model parameters are: (1) a standard horizontal grid resolution of 64 longitude by 48 latitude and a vertical layer of 49, (2) initial surface pressure 610 Pa, (3) 38 tracers, (4) obliquity (25.19°) and eccentricity (0.0934). The red plus symbol is the Zhurong landing site. **b**,**e**,**f**, The model for the formation of the bright barchans (a) in **b** and **e** and dark sands (b, c) in **b** and **f** accumulation under two different windblown sand activity regimes. The blue and green arrows respectively indicate the wind directions of the two wind field regimes. The blue line shows the wind-erosion trace in **e** and **f**. The green line shows the windblown linear ridges in **f**.

These documented Zhurong landing zone aeolian landforms permit the characterization of wind fields, with the ubiquitous bright barchans and dark longitudinal dunes representing two contrasting windblown sediment regimes (Fig. 3b,e,f). The earlier-stage regime is characterized by isolated bright barchans with encrusted surfaces, interpreted as representing a relatively stable, weak wind field (roughly 12.5° azimuth predominant wind direction) and low sediment sources. The later-staged regime is characterized by dark longitudinal dunes; this regime modified, partly degraded and eroded, and then partly covered bright barchans, revealing reorientation of the wind field (strong winds mainly from roughly 300° azimuth) averaging out any local variability. The more than 70° difference in the two regime wind directions implies a significant change in atmospheric circulation in low–mid-latitude Utopia Planitia, and perhaps globally. Dark sediment accumulations are smaller than bright barchans, suggesting more limited sediment sources, indicating either a relatively short duration episode of the most recent wind regime or to a climatic condition insufficient for sand mobility.

The encrusted bright barchan surfaces and the agglomerated particles on dark sand accumulations are similar to ubiquitous regolith induration observations^25^. It seems plausible that they formed as duricrusts from slow chemical weathering and/or salt formation^25,27,28^; the dust induration is also similar to that seen in lithified dune occurrences^29–31^. When small amounts of water vapour interact with porous surface regolith on daily or longer timescales, very thin transient aqueous films are produced on grain surfaces that enable chemical interactions^32–34^. Mars Climate Station (MCS) observations and GCM simulations^35,36^ show that condition conducive to the presence of water frost and liquid brines were available in the Zhurong exploration region (Methods and Extended Data Fig. 9). Therefore, participation of brines may be the cause of the agglomeration of dark sand. SWIR data also show the presence of hydrous sulfate and chloride in the dunes. Thus, sulfate and chloride are the most likely materials causing agglomeration. Bright barchan surface crusts may also indicate extra brine involvement and a longer-duration weathering process.

On the basis of aeolian features and the morphology of the dunes documented here, the Zhurong zone is interpreted to have experienced two main climatic stages, marked by changes in wind direction regimes. The early stage was characterized by a north-east (azimuth roughly 12.5°) wind field and development of bright barchans. Under changing temperature and humidity conditions, bright barchans ceased actively forming and crusts and cracks developed on their surfaces. The later stage was marked by a north-west (azimuth roughly 300°) wind field, during which the duricrusted bright barchan surfaces were eroded, sediment agglomeration occurred and smaller dark dunes accumulated.

What is the age and duration of these two windblown regimes? We counted 38 craters superimposed on the 2,262 bright barchans and used impact crater size-frequency distribution (CSFD) methods to estimate absolute model ages (AMA) of the Zhurong zone bright barchans. Recognizing the caveats in interpreting small count areas^37^, our results show a bright barchan population AMA between 2 and 0.4 Myr, supporting the interpretation that the earlier regime ceased in line with end of ice age (Methods, Extended Data Fig. 8 and Supplementary Table 4). Previous studies have reported TAR ages from 3.0 to 0.1 Myr (refs. ^20,38–40^); these similar ages support a contemporary windblown sand deposit age for the formation of Mars bright barchans. Superposition of dark longitudinal dunes, sand ridges and surficial-coated sands on bright barchans reveals that the second regime was active subsequent to end of ice age, in the modern climate regime.

## Correlation with global climate change

**Fig. 4:**
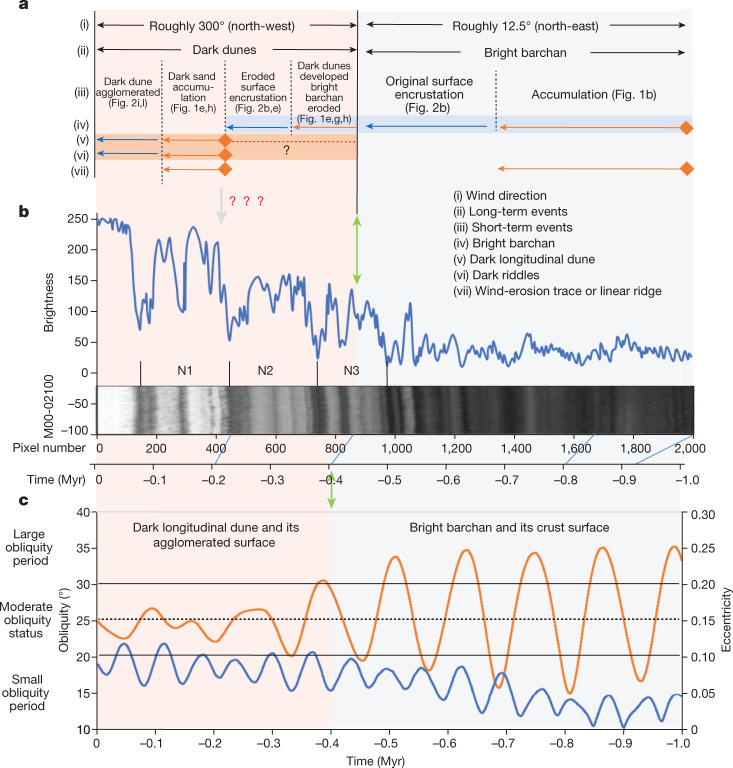
Comparison of the paleoclimate events inferred from the dunes with the grey level of North pole stratified strata and orbital parameters of Mars. **a**, The sequence of paleoclimate events inferred from the dunes (orange lines and labels represent sand accumulation events and dark blue lines and labels represent dune surface encrustation or agglomeration, respectively, and the dotted line with question marks (?) indicates unclear). **b**, The brightness profile versus pixel number for a section of the Mars Orbiter Camera image M00-02100 (bottom) on 13 April 1999 (*L*_s_ equal to roughly 123°), at latitude 86.48°. There are three similar cycles (N1, N2, N3) between pixels 100 and 1,000. The time axis was established by orbital tuning^7^. **c**, Martian obliquity and eccentricity curves^3^ over the last 1 Myr. The orange line shows the curve of obliquity and the blue line shows the curve of eccentricity.

Orbital and spin-axis parameters of Mars (inclination, eccentricity, precession) play big roles in global climate change^3^. The potential effects of these variations on recent ice ages led to the proposal from orbital data that a geologically recent ice age occurred (from roughly 2.1 to 0.4 Myr) and that after roughly 0.4 Myr, Mars entered an interglacial period^1^ (Fig. 4c). These results strongly indicate that obliquity changes drive global climatic events^41^. In addition, this global climatic transition is predicted to have occurred at a time that we independently from find both stratigraphic and preliminary crater-count data to be simultaneous with the immobilization of bright barchans in the Utopia Planitia Zhurong region. If these two events are time-correlative, then we can further test this interpretation by searching for corroborating evidence in the North polar cap.

The alternating bright and dark layers forming the North polar cap have long been considered to be due to climate changes caused by variation in orbital parameters^42–45^. A North polar cap chronological sequence was reconstructed^7^ using a layered terrain brightness profile, orbital parameters and north pole summer insolation at summer equinox (*L*_S_ = 90°) (*L*_s_, solar longitude; the Mars–Sun angle measured from the Northern Hemisphere spring equinox, where *L*_s_ = 0). High brightness values for the upper three cycles showed clear contrast to the underlying low-brightness part, with the abrupt brightness increase occurring at roughly 0.4 Myr (Fig. 4b). We thus find a second, independent, line of evidence for a change in climatic regime that seems to be synchronous with the immobilization of bright barchans in southern Utopia Planitia. These three lines of evidence (Zhurong Utopia results reported here, global geomorphic evidence for an ice age-interglacial transition^1^ and recent North polar cap stratigraphy^7^) all indicate that a global climate change event occurred, from Martian polar regions to low-mid-latitudes. We now explore how detailed Zhurong findings can assist further in assessing and understanding the nature of this global climate transition.

## Influence of obliquity adjustments

Between roughly 0.4 and 2.1 Myr, obliquity amplitude oscillated^8^ between roughly 15° and 35°. At high obliquity, mean wind direction in the Zhurong zone (roughly 25° N) is north-east, characterized by deep Hadley circulation^10^. Under this stable, continuous and weak northeastern wind field, small barchans can readily form (Fig. 3b,e). This formation mechanism matches the characteristics of bright dunes mainly distributed at low-mid-latitudes^14^. A Zhurong zone dust deposition period due to water cycle enhancement^46^ is also predicted, with dust and brine activity conditions promoting bright barchan surface cementation. When obliquity amplitude fell to values less than 25°, decreasing temperatures could further promote bright barchan cementation and crust formation, potentially increasing dune albedo. We thus interpret Zhurong bright barchans as forming during the high obliquity period, and degradation and cementation occurs at lower obliquity, exiting the earlier climate regime in both a well-preserved and partly indurated condition.

After roughly 0.4 Myr ago, obliquity amplitude decreased to below 30°, remaining relatively stable^3,7^, varying from 22° to 26°, a period described as interglacial^1^. At the Zhurong zone (roughly 25° N), the influence of northeastern Hadley circulation was weak and water ice in the shallow subsurface was thermodynamically unstable^47–49^. The porous regolith gas diffusion rate was reduced, preventing local surfaces from intense weathering and degradation and weakening winds that are capable of transporting sediments.

GCM model results (Fig. 3a) indicate that when obliquity exceeded 25°, the annual mean wind direction was still north-east; however, in Northern Hemisphere solstice seasons, trade winds cross the Equator, bend to the east and form a low-level eastward jet in the summer subtropics^50^. MCS measurements and GCM simulations show that north-west landing area winds may occur at *L*_s_ = 120–150° (Methods, Fig. 3c,d and Extended Data Fig. 10). Although the north-west wind speed measured by MCS and simulated by GCM did not meet the minimum threshold wind speed for the initiation of sand grain movement on the Martian surface, Battalio and Wang^51^ observed that five dust storms passed through the Zhurong landing site, which travelled towards the south-east, during the 1999–2014 period. The wind field becomes stronger and more unstable at this time, providing conditions we interpret to be sufficient to form the dark longitudinal dunes superposed on bright barchans (Fig. 3b,f). During the most recent regime (obliquity below 25°), the lower-latitude Zhurong region lacks ice and dust sediments typical of the medium-higher latitudes^1^; surface hardening, chemical weathering and salt formation are predicted to be reduced, resulting in agglomerated particles on the dark dunes.

During the current low-obliquity-amplitude regime, small climate fluctuations occur on 10,000-year scales^3^. There are two periods during which obliquity exceeds 25°, comparing favourably with the 2.5 cycles observed following the bright barchan regime (Fig. 4a). Owing to the lack of accurate age constraints, it is difficult to correlate precisely the individual polar deposit brightness changes with a specific climate^7,45^. Nonetheless, this suggests that the climate is likely to have undergone small-amplitude variations following bright-sand activity cessation.

The roughly 0.4 Myr obliquity amplitude decrease^3,7^ led to a distinctive transformation of Martian global climate from glacial to interglacial^1^. This epoch-making change in climate regime appears to have been captured in detail by the dune morphology, orientation, physical properties and stratigraphy in the Zhurong landing area. The bright barchans and dark longitudinal dunes together illustrate that the predominant wind field underwent a roughly 70° change. Even though nearly all Martian GCM models show that the average predominant wind direction and annual wind direction did not change (Fig. 3a), it is clear from the Zhurong data that seasonal variations (not captured by the averaged GCM data) may indeed have changed. The occurrence and strengthening of north-west wind in the younger regime can be best explained by seasonal wind direction variation and related variations in atmospheric dust^10^ (Fig. 3c,d). The formation mechanism of bright sand dunes and dark sand accumulation proposed here is derived from detailed in situ data that complements and supplements the information provided by the GCMs, potentially providing new insights into finer-scale (seasonal) wind direction changes.

The Tianwen-1 and Zhurong results underline the strength of comprehensive and well-coordinated payload packages permitting multidisciplinary in situ analyses of regional to global problems in climate evolution. The results demonstrate that well-equipped rovers targeted to key locations^1,2,4,5^ can provide essential data in reconstructing the climate history of Mars and supplementing and potentially improving climate GCMs.

## Methods

### Investigation and instruments

The previous rover-scale exploration by Sojourner/Ares Vallis^52^, Spirit/Gusev^53^, Opportunity/ Meridiani^54,55^, Curiosity/Gale^56–59^ and Perseverance/Jezero^60–62^, provide critical information on recent climate-related processes^63,64^ and can also provide links to wind directions^65^. surface alteration environments^66^ and aeolian processes^67,68^. China’s Tianwen-1/Zhurong rover made critical remote sensing and in situ observations of aeolian features in the Zhurong landing zone.

The high-resolution imaging camera^69^ (HiRIC) is onboard the Tianwen-1(TW-1) orbiter, which uses an off-axis three-mirror astigmatic optical system with a focal length of 4,640 mm. Three time delay and integration charge coupled device detectors are all set on the imaging plane to achieve the push-broom imaging. Each time delay and integration charge coupled device has an array of 6,144 panchromatic band pixels lined perpendicularly to the direction of the push-broom, with a pixel size of 8.75 × 8.75 μm. Topographic data products with 3.5 m spatial resolution were derived on the basis of HiRIC image data (0.7 m per pixel) collected during the parking-orbit period of the TW-1 orbiter^70^.

The navigation and terrain cameras^71^ (NaTeCam) are binocular stereo cameras mounted on the Zhurong Mars Rover, 1.8 m above the Martian surface, with a stereo baseline of 270 mm. NaTeCam is able to achieve 1.2 mm per pixel resolution when imaging a target at 3 m distance. NaTeCam images were processed through dark current correction, non-uniformity correction, absolute radiometric correction and colour correction. NaTeCam has a yaw angle range of ±178.5° and a pitch angle range from −70° to 90°, which enables ring-shot stereo imaging of the terrain surrounding the rover for topographic reconstruction.

The multispectral camera^72^ (MSCam) is mounted on the rover’s mast. MSCam has a focal length of 37.5 mm, with 24° diagonal field of view and 0.15 mrad angular resolution. The MSCam uses a complementary metal-oxide-semiconductor imaging detector to produce a 2,048 × 2,048 pixel image. Owing to the constraints of relay data transmission, the full image is split into 8 × 8 small windows and every subimage is composed of 256 × 256 pixels. In the default operation mode, only several windows (3 × 2 windows or 2 × 3 windows) of most interest within the full image will be uncompressed and downlinked. The 2,048 × 2,048 full image will only be downlinked in the compressed mode. Its multispectral channels are produced through a filter wheel composed of eight narrowband spectral filters (480, 525, 650, 700, 800, 900, 950 and 1,000 nm) and a broadband solar observation filter (480–600 nm). MSCam images were processed through dark current removal, flat field correction and radiometric calibration to convert observed digital number into physical radiance, and then derived reflectance from the radiance using onboard reference data of the MSCam calibration target^73^. The red, green and blue colours were calculated by the colour fit method^74^ using the eight narrowband spectral image data of MSCam.

The MarSCoDe^75^, which consists of a LIBS, a SWIR spectrometer and a micro-imager, is applied to probe targets on the Martian surface. MarSCoDe provides functions such as Martian surface elemental composition analysis and mineral identification through collecting active LIBS spectra (240–850 nm) and passive SWIR spectra (850–2,400 nm).

The MCS^76^ mounted on the rover can measure local temperature, pressure, wind and sound on the Martian surface.

The High-Resolution Imaging Science Experiment (HiRISE) mounted on Mars Reconnaissance Orbiter provides 0.25 to 1.3 m spatial resolution images^77–80^. Here, we used a HiRISE image (roughly 0.25 m per pixel) that covered the Zhurong landing area for dune analysis (Hires_ESP_069665_2055_RED.JP2).

### Dune morphological features from remote sensing images

Using orbital images from HiRISE and HiRIC, the boundaries of 2,262 dunes were extracted from the plains around the landing site of the Zhurong rover, which covered 7 km from north to south and 5 km from west to east, after excluding disturbed regions such as impact craters, troughs and so on (Extended Data Fig. 1a). These dunes in the landing zone are sparsely distributed, disconnected and isolated, accounting for only 1.4% of the total area of the region. These dunes around the landing site are mainly represented by a ‘bright barchan-like’ shape, formed by a unidirectional wind perpendicular to the crestline. Statistics of dune morphology were extracted from their boundaries, including area of the dune boundary enclosure, the crest-ridge width (*W*), the ‘down wind’ length (the dimension following the inferred wind direction) (*L*), crestline length (r*L*), sinuosity (*W*/r*L*), crest to crest wavelength or spacing (λ) and wind direction indicated by dunes (Extended Data Fig. 1b). In this paper, following the geographic convention, our wind directions were given in azimuths towards which the wind blows. The azimuth of the wind direction is 0° from the north increasing clockwise to 360°.

The statistical results showed that the wind directions indicated by 95% of the bright barchans are mainly distributed in the range between 341° and 27°. The predominant wind direction was from the north-east with an azimuth of 12.5°, as illustrated in Fig. 1c and Extended Data Fig. 1c. The areas of bright barchans (95%) are between 24 and 699 m^2^ and the most common dune area is roughly 100 m^2^. The frequency decreases exponentially with increasing area. The crest-ridge width (*W*) of 95% of the dunes is in the 11–72 m range, crestline length (rL) also between 11 and 72 m and sinuosity between 0.8 and 1.0, increasing exponentially to 1. The down wind length (*L*) ranges from 2 to 13 m, and the crest to crest wavelength (*λ*) is around 149 m (Extended Data Fig. 1d–g). On the basis of these basic measurements, we defined plan view aspect ratio *a*, where *a* = *W*/*L*, and inter-bedform spacing *s*, where *s* = *λ*/*L* (ref. ^14^). In general^32^, TARs have a high aspect ratio (*a* > 6) and sometimes as high as 15. However, the *a* value of those in the Zhurong landing zone is slightly smaller, only about 5–6, which corresponds to small bright barchans. TARs with *s* = 1 were referred to as saturated, *s* < 2 as being closely spaced and those with higher *s* values as being discontinuous or widely spaced. The bright barchans in the Zhurong landing zone have higher values (*s* > 11), indicating that they are discontinuous or widely spaced.

Among the 2,262 bright barchans, 1,096 (roughly 48%) modified dunes can be identified from HiRIC and HiRISE images. Longitudinal dunes are developed on their western horns and a tip tail formed on their eastern horns. Among these, 500 significantly modified dunes (Extended Data Fig. 2a–c) were selected for detailed analysis. The orientations of longitudinal dunes and tip tails on the western and eastern horns of these selected dunes were analysed. The minimum, maximum and average orientations of the longitudinal dunes are 290.4°, 318.4° and 301.4°, respectively. The minimum, maximum and average orientations of the tip tails are 282.6°, 315.6° and 300.8°, respectively (Supplementary Table 1). The two orientations are similar, indicating that the eastern and western horns of the bright barchans were modified under a wind field with azimuth of roughly 300°. The west horns against the wind direction were shortened and thickened by wind denudation. The trend of eastern horns, however, was aligned with the wind direction, and therefore the horns were forced to stretch out and formed an eyebrow-like tip tail along the wind direction. Under the influence of wind, an east–west asymmetric morphology of the dune was formed.

### Dune morphological features observed by Zhurong rover

Five bright barchans (dunes 1–5) were investigated in situ (Fig. 1). By using ContextCapture Master and Photoscan software, the three-dimensional models (in .obj format), digital elevation models and digital orthophoto maps of these five dunes were processed on the basis of the stereo images captured by NaTeCam.

The morphometric parameters of these dunes have been measured using 3d Max. The areas of bright barchans are in the 131–435 m^2^ range. The crestline length (rL) range is 29.0–66.1 m, the down wind length (*L*) 8.0–11.7 m. with heights of 0.5–1.0 m, representing individual examples of medium to large dunes around the landing site. The north slopes and south slopes of the five dunes range from 7.9 to 11.9° and 9.5 to 13.9°, respectively, being far lower than the common angle of repose around 30°–35°, which also indicates that these dunes have been modified (Extended Data Fig. 3a and Supplementary Table 2).

Previous studies reported on the morphology of TARs with high albedo, documenting an average crestline length of 215 m, height in the 1.5–5.7 m range and wavelengths in the 38–40 m range^34,81,82^. The bright barchans around the Zhurong landing site are relatively short, small and show a sparse distribution in comparison.

The digital orthophoto maps and three-dimensional models of dunes 2 and 5 were measured using ArcMap and 3d Max. The morphometric parameters of five and three longitudinal dunes, respectively, located in their west horns were obtained. These longitudinal dunes were all arranged roughly in parallel. Their trends were oriented between 278.7° and 295.9° for dune 2 and 282.4° and 294.7° for dune 5. The length of longitudinal dunes was 4.8–10.0 m long for dune 2 and about 4.0 m for dune 5. Their spacings varied from 1.4 to 3.0 m for dune 2 and 0.7–1.3 m for dune 5. The slope of the south side and north side of each longitudinal dune was not symmetric as shown by the topographic profiles (Extended Data Figs. 3b,c and 4a,b). There was some variation in the orientations of these longitudinal dunes, indicating that the wind field was variable when these longitudinal dunes were formed.

Some transverse ripples accumulated on interdune depression of longitudinal dunes due to the weakened wind strength in the down wind direction. Their orientations are perpendicular to the longitudinal dunes and have spacings of 0.3–1.0 m for dune 2 (Extended Data Fig. 3d,e) and 0.3–0.6 m for dune 5 (Extended Data Fig. 4a).

### Surface properties of bright and dark sands

Several observations were made on the bright and dark sand regions on barchan surfaces by MarSCoDe and MSCam as denoted in Fig. 2a–n and Extended Data Fig. 5c,d.

Images from MSCam showed that the dark sand surfaces contained abundant agglomerated particles (roughly 3–15 mm in diameter), resembling a rough surface (Fig. 2d,i,l–n), whereas the bright sand surfaces were shown to have relatively smoothed crusts (Fig. 2b,d–f) with cracks (Fig. 2g,h,j,k). Most cracks were polygons with 3–7 sides with a length range from 0.3 to 13.5 cm (average 4.8 cm) and inner-angles ranging from 20 to 190° (average 110°). In addition, some parallel cracks developed on the denudation surfaces of the bright barchans, with orientations paralleling both the contour line and that of the longitudinal dunes. Dark sands filled in the grooves of the parallel cracks. The spacing between parallel cracks was measured at eight regions (Extended Data Fig. 3b,f), and ranges from 2 to 4 cm.

The images from MarSCoDe micro-imager showed that both bright and dark sand surfaces were shattered into a hole-like crater with a diameter of roughly 4 mm and fine powder with 100–300 μm in diameter after being hit using LIBS laser shots (Fig. 2f,n and Extended Data Fig. 5c,d), indicating that they represented cementation of small sand grains formed under some unknown mechanism.

The roughness of dune surface was analysed by computing the dissimilarity property of the grey level co-occurrence matrix (GLCM)^83^ derived from dune surface images. In total, ten image regions of dune surfaces were clipped from MSCam data and each image region was 200 × 200 pixels in size and had a uniform spatial resolution of 300 μm per pixel after resampling. The dissimilarity of three bright sand image regions ranged from 13.8 to 21.6 and averaged 17.0, whereas the dissimilarity of seven dark sand image regions ranged from 25.9 to 38.2 and averaged 30.4, nearly twice greater compared to bright sand; this indicates a significantly rougher surface on the dark sand surface.

**Extended Data Fig. 6:**
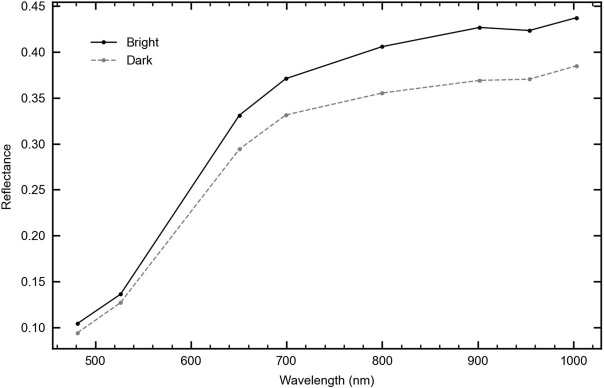
Reflectance factors (RF) of bright and dark sands. The black solid line and dashed line represent the RFs of bright and dark sands, respectively.

At adjacent locations, the average value of reflectance increases in bright sand relative to dark sand in eight bands of MSCam and is greater than 15% (Extended Data Fig. 6).

### Compositions of bright and dark sand surfaces

Reflectance spectra of dunes 1–3, from 850 to 2,400 nm, were derived from corresponding SWIR radiance data through wavelength and absolute reflectance calibrations using an onboard calibration target (a white board) and ground laboratory data^84^. The reflectance spectra were further smoothed by a sliding 15-pixel average filter and compared to typical Martian mineral spectrum recorded by standard mineral spectra in the RELAB spectrum library^85^.

**Extended Data Fig. 7:**
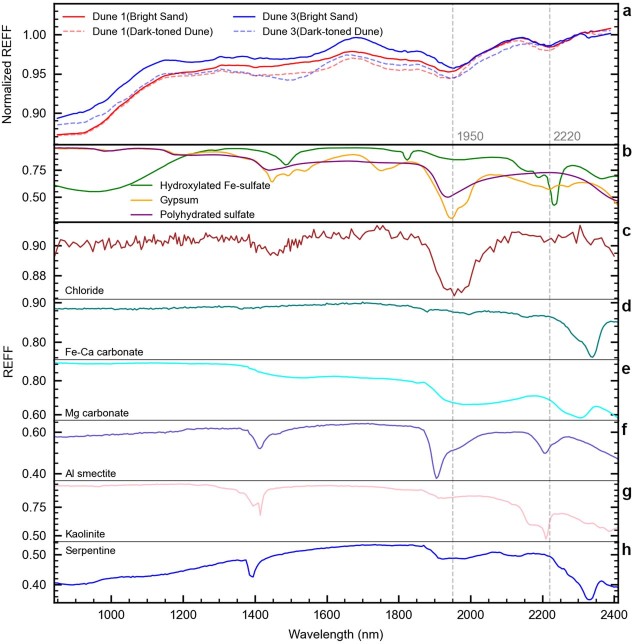
Comparison of the spectra of bright and dark sands with minerals recorded by standard mineral spectrum of RELAB. **a**, SWIR spectra of Dune 1 and Dune 3, red solid and dashed lines are respectively bright and dark sands SWIR spectra of Dune 1, while those of Dune 3 in blue. **b**,**c**, are respectively RELAB spectra of hydroxylated Fe-sulfate (Fe(OH) SO_4_, the green line), gypsum (CaSO_4_·2H_2_O, the orange line), polyhydrated sulfate (MgSO_4_·nH_2_O, the purple line) and chlorides (the brown line). **d**–**h**, are laboratory spectra of the following minerals: (Ca,Fe)CO_3_, MgCO_3_, (Ca, Na)_0.3−0.5_(Al, Mg)_2_(Si, Al)_4_O_10_(OH)_2_·nH^2^O, Al_2_(Si_2_O_5_)(OH)_4_, (Mg, Fe)_3_Si_2_O_5_(OH)_4_. The reflectance factor (REFF) represents the ratio of the reflectance of the surface to that of a perfectly diffuse surface under the same conditions of illumination and measurement. The radiance factor (RADF, I/F) is the ratio of the bidirectional reflectance of a surface to that of a perfectly diffuse surface illuminated at I = 0. Normalized REFF is obtained by normalizing REFF to 1.0 at 2300 nm.

The reflectance spectra were normalized to 1.0 reflectance at 2,300 nm. The spectral features of bright and dark sand both showed strong absorption features around 1.95 and 2.22 μm (Extended Data Fig. 7a). The parabola-fitting results for 1.95 and 2.22 μm absorption-band positions showed that the spectral features are similar to that of hydroxylated Fe-sulfate (Fe(OH) SO_4_), gypsum (CaSO_4_·2H_2_O), polyhydrated sulfate (MgSO_4_·nH_2_O) and chloride ((K, Na, Mg)Cl) (Extended Data Fig. 7b,c), but significantly differed from that of carbonates or phyllosilicates (Extended Data Fig. 7d–h). These observations indicate the existence of salts that are dominated by hydrated sulfates and chlorides in the compositions of sand particles.

A natural gradient boosting probabilistic prediction model (NGBoost)^86^ was established using MarSCoDe laboratory data, which had been used to quantitatively predict the main element compositions of bright and dark sands. The prediction root mean squared error (wt%) for the results of seven calibration targets was 0.11–3.49%. Main oxide concentrations of bright sand and dark sand are shown in Supplementary Table 3. The predicted total main oxides sum turned out to be below 100%. Among them, bright sand is 85.78 ± 3.80% and dark sand is 90.59 ± 3.41%. The undetectable part may be related to S, H, Cl and so on. In the LIBS spectrum, the H line at 656.468 nm could be clearly recognized. S and Cl signals in the LIBS spectrum were not detected, largely due to its weak emission below the noise level of MarSCoDe.

The composition of bright and dark sands was similar to that of average Martian soil^87^, close to basalt composition. The bright sands contain more Mg and more undetectable parts than the dark sands. Therefore, the bright sands may have more hydrated sulfate or chlorides than the dark sands.

### Estimating AMA of the bright barchan dunes

**Extended Data Fig. 8:**
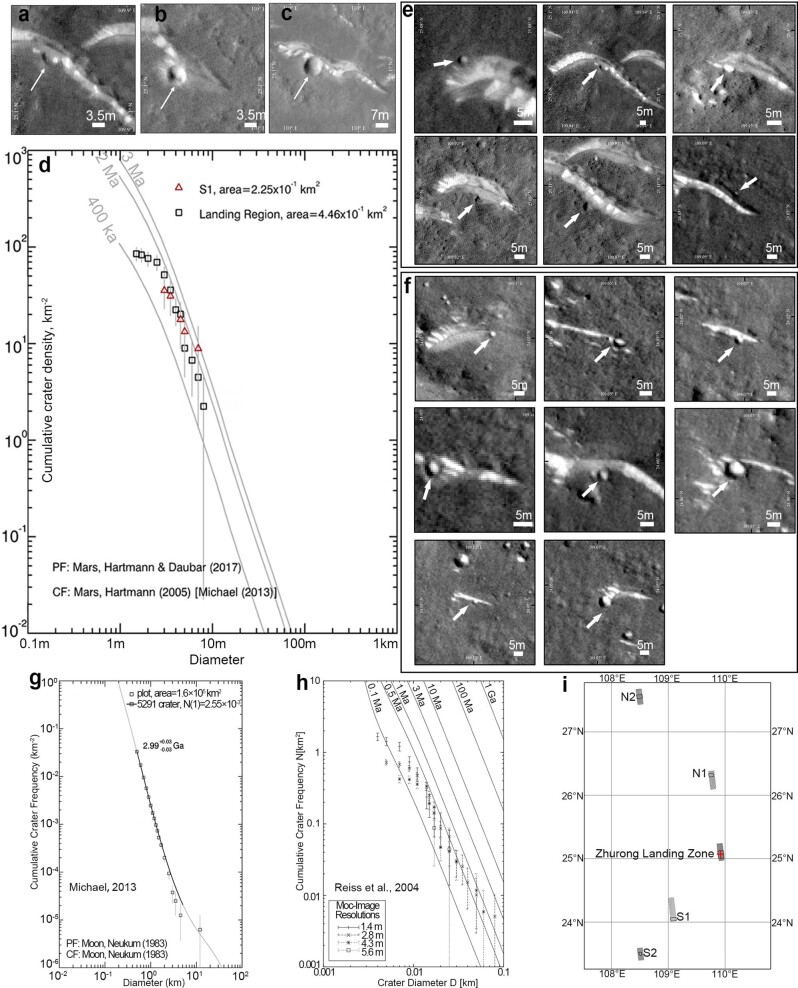
Age dating of bright barchans. **a**, is a crater mantled by dune (white arrow). This type of crater was excluded during dating. **b**,**c**, are craters that are superimposed on the dunes. The images are extracted from HiRISE images. **d**, Crater size-frequency distribution of dunes in the Zhurong landing zone and S1 region. The black rectangle represents the results of the Zhurong landing zone. The red triangle represents the results of S1 located in the southern region of the Zhurong landing zone. Error bars are 1*σ* s.e. **e**, craters which may easily be misjudged and were excluded during dunes dating. **f**, the mapped craters in S1 region. **g**, the best-fit age determination method commonly used for planetary surfaces (Michael, 2013). **h**, the commonly used presentation of age determination for barchan dunes (Reiss et al., 2004). **i**, the spatial distribution of areas studied for bright barchan dating. Red + is the Zhurong landing site, and black boxes are the 4 flat areas selected for dune dating.

The CSFD method was used to acquire estimated AMAs of the bright barchans within the Zhurong landing site. On the basis of the derived 2,262 dunes, the CraterTools^88^ on the ArcGIS platform was used to correctly extract craters superimposed on the dunes (Extended Data Fig. 8b,c). During the mapping of craters, we were very careful to avoid obvious crater chains and clusters especially near large primary craters, to exclude secondary craters. We undertook a comprehensive search for any primary craters that might have polluted our count areas with secondary craters and found no supporting evidence for this potential effect. We excluded craters mantled by the dunes (Extended Data Fig. 8a). Through cross-checking by several experts, we more cautiously eliminated a few impact craters (Extended Data Fig. 8e) that may easily be misjudged.

For the age dating of Martian surface based on CSFD, the chronology function model^89^ and the impact crater production function model^90^ is offered, showing our current best-estimate production size-frequency distribution curves down to *D* = 1 m. With the improvement of image resolution, metre-scale diameters craters were used for dune dating^40,91^. In this study, 38 effective craters (with diameters of 1.6–8.6 m) were identified (Supplementary Table 5). Considering that craters degrade more quickly on the surface of Martian barchan dunes, the dating error may be larger than that of rock surfaces. Instead of fitting a dating curve to give a certain model age value with error (±uncertainty) (Extended Data Fig. 8g), all age data values and their error bars are constrained between fitting upper and lower isochrons (Extended Data Fig. 8h) to help constrain the age of the dunes. All age data values and their error bars of bright barchans within the Zhurong landing site range from roughly 0.4 to 2 Myr (Extended Data Fig. 8d and Supplementary Table 4). To further constrain the rationality of the CSFD dating results, we also used the crater retention age method mentioned in Reiss et al.^39^ to estimate the retention age of the largest crater (roughly 8.6 m in diameter) on dunes, acquiring a roughly 1.7 Ma as rough upper limit of the dune stabilization age, which is roughly consistent with the results obtained by CSFD.

Warner et al.^37^ have clearly shown that interpreting AMAs from impact CSFD data is made much more robust by (1) larger surface count areas and (2) larger numbers of counted craters, and the clear exclusion of secondary craters in the counts. With these caveats concerning the small area available for analysis^37^, we tentatively interpret the time of bright barchan activity to have ceased in line with end of ice age, and the uncertainties emphasize the need for many Mars sample return missions to increase the precision of an age estimate on Mars.

With the Zhurong landing area as the centre and extending roughly 2–3° each to the north and south, four flat areas were selected for dune dating (Extended Data Fig. 8i). Areas characterized by obvious undulating terrain (for example, large impact craters and troughs), which may influence the local winds, were avoided. Supplementary Table 4 shows the dating information of these areas, which vary from 7 to 19 km^2^ in areal extent, with the number of dunes ranging from 26 to 1,027. Northwards from the Zhurong landing zone, the dunes in N1 and N2 are less widely distributed, and the number of superimposed impact craters is very few (only one or even none in Supplementary Table 4). It is therefore difficult to date dune activity in N1 and N2. Southwards from the Zhurong landing zone, the number of superimposed impact craters in region S1 is eight (Extended Data Fig. 8f and Supplementary Table 6) and in region S2 is two. Noting the caveats described above, the CSFD curves (Extended Data Fig. 8d) tentatively indicate that the cessation time of bright barchans activity is similar to that of bright barchans in the Zhurong landing zone.

### Surface temperature and water vapour partial pressure at the Zhurong landing site

**Extended Data Fig. 9:**
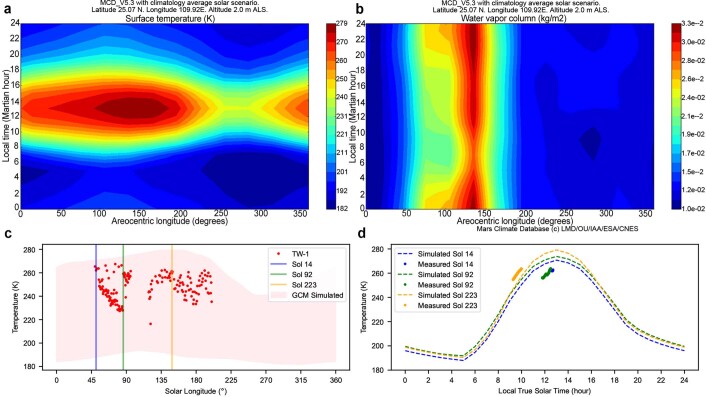
The surface temperature and water vapor column abundance at the Zhurong landing site. **a**,**b**, The GCM simulated surface temperature and water vapor column abundance of the average solar scenario. **c**, Comparison between simulated temperature and measured temperature by MCS. **d**, Partial enlarged view at the vertical line in **c**. The enlarged view reflects the daily variation of temperature.

The surface temperature and water vapour partial pressure of the average solar scenario at the Zhurong landing site were simulated by using a GCM^35,36^. The simulated results are 182–279 K and 0.04–0.12 Pa (Extended Data Fig. 9a,b), respectively. The Modified Tetens equation (1)^92^, which described the relationship between temperature and saturated water vapour pressure, was used to calculate the water frost temperature.

1 $$E=6.112\times {{\rm{e}}}^{17.67\times \frac{t}{t+243.5}}$$

where *t* = *T* - 273.15 in °C, *T* is absolute temperature in Kelvin, and *E* is the saturated water vapour partial pressure in hPa. Let *E* = 0.0004 hPa or *E* = 0.0012 hPa, and substitute into equation (1), to obtain the *t* = −86 °C (187 K) or −79 °C (194 K). Because saturated water vapour pressure should be greater than or equal to water vapour partial pressure, the water frost temperature of the Zhurong landing site must be greater than or equal to 187–194 K according to equation (1).

A global map of H_2_O frost temperature was calculated using seasonally averaged Thermal Emission Spectrometer (TES) water vapour column abundance. According to the map, water frost would be present at low latitudes of Mars, and the water frost temperature at the Zhurong landing site^93^ was 197–199 K.

For the frost mixed with salt, when the temperature exceeds the eutectic temperature of the mixture, the frost melts and forms liquid saline water. Assuming the soil of the Zhurong landing site contains a mixture of MgCl_2_ and MgSO_4_, the eutectic temperature^94^ would be 238 K. According to the GCM simulation, the surface temperature of the Zhurong landing site could cover a range of 182–279 K. The simulation results were in good agreement with the measurement results captured by the MCS onboard the Zhurong rover (Extended Data Fig. 9c,d).

Compared with the water frost point and eutectic temperature of liquid brine, the Zhurong landing zone should be characterized by conditions in a *L*_s_ season 225 and 240, in which the water vapour could have been emplaced as frost and the frost could also melt into liquid brine.

### Surface pressure, wind directions and magnitudes at the Zhurong landing site

The surface pressure, wind directions and magnitudes of the average solar flux at the Zhurong landing site were simulated by using a GCM^35,36^. During the in situ investigation, MCS onboard the Zhurong rover worked continuously for 5–105 min near noon on Mars, and measured local surface pressure and wind field.

**Extended Data Fig. 10:**
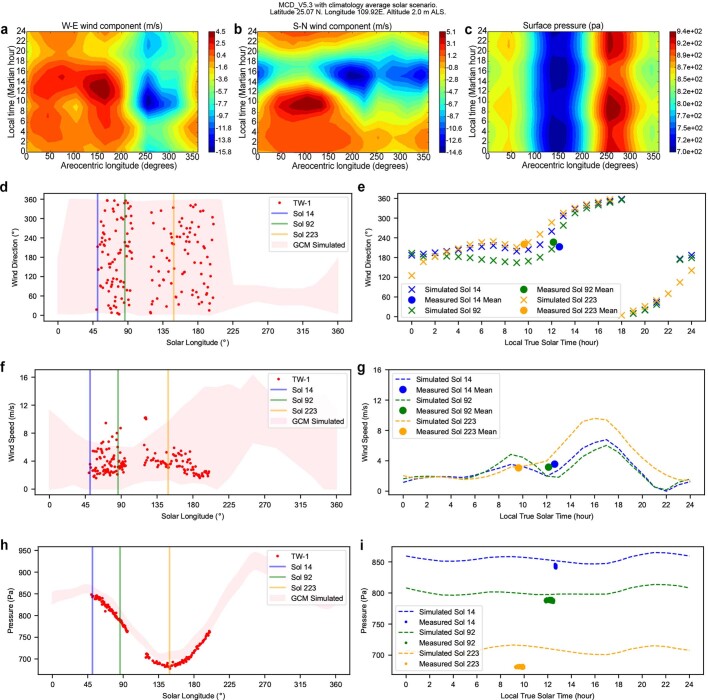
The surface wind directions, wind magnitudes and pressure at the Zhurong landing site. **a**,**b**, the GCM simulated W-E and S-N wind of average solar scenario. **c**, the GCM simulated pressure of average solar scenario. **d**,**e**, Comparison between simulated wind direction and measured wind direction by MCS. **f**,**g**, Comparison between simulated wind speed and measured daily average wind magnitudes by MCS. **h**,**i**, Comparison between simulated pressure and measured pressure by MCS. **e**,**g**,**i**, are the partial enlarged views at the vertical lines of **d**,**f**,**h**, respectively. These enlarged views reflect the daily variation of wind directions, wind magnitudes and pressure.

The average from the daily MCS measurement data agrees with the simulated daily variation of wind directions, wind magnitudes and pressure (for example, Sol14/Ls51.0, Sol92/Ls86.1 and Sol1223/Ls148.9) (Extended Data Fig. 10e,g,i).

From Extended Data Fig. 10d,f,h, the GCM simulated results and MCS measured data in terms of seasonal changes were also consistent.

In addition, the wind speed conditions to form dunes should be at least 15.07–24.09 m s^−1^ (ref. ^95^). From the GCM simulations in Extended Data Fig. 10d,f, it is clear that during Ls225°–270°, the wind direction is the north-east wind (azimuth 30–90°) and the wind speed meets the above-mentioned threshold, which is favourable for barchan dunes deposition. During recent ice ages at higher obliquity cases, the north-east winds would be stronger and more favourable for the formation of barchan due to the deep Hadley circulation, whereas the dark longitudinal dunes were formed under north-west winds. Although the GCM simulations showed the presence of north-west winds at the Zhurong landing site, their simulated wind speeds did not reach the above-mentioned threshold. However, there were five dust storms that passed through the Zhurong landing site, which travelled towards the south-east, during the 1999–2014 period^51^. These dust storms from the north-west were favourable for the modification of barchan dunes and the development of dark longitudinal dunes.

## Online content

Any methods, additional references, Nature Portfolio reporting summaries, source data, extended data, supplementary information, acknowledgements, peer review information; details of author contributions and competing interests; and statements of data and code availability are available at 10.1038/s41586-023-06206-1.

## Supplementary information

Supplementary Tables This file contains Supplementary Tables 1–6.

## Data Availability

The Tianwen-1 mission and Zhurong rover data used in this work is processed and produced by the GRAS of China’s Lunar and Planetary Exploration Program, provided by the CNSA. All data in the main text and the supplementary materials are available at 10.12350/CLPDS.GRAS.TW1.AD-MarsDunes.v202304.
