# Supplementary material for: Martian dunes indicative of wind regime shift in line with end of ice age
Source: Nature. 2023 Jul 5;620(7973):303–9. doi: 10.1038/s41586-023-06206-1 (PMC10412455; doi:10.1038/s41586-023-06206-1)
Supplement: Supplementary file 1 — This file contains Supplementary Tables 1–6. [file 41586_2023_6206_MOESM1_ESM.pdf]

nature portfolio

https://doi.org/10.1038/s41586-023-06206-1

Supplementary information

Martian dunes indicative of wind regime shift in line with end of ice age

In the format provided by the authors and unedited

Supplementary Table 1 | Statistical information on wind direction documented by aeolian features and landforms

Wind field	Type of aeolian features and landforms	Wind direction (minimum-maximum)
Early-stage	Bright Barchans	306.9°-52.0°
	Wind-erosion trace on soil Sol31 NaTeCam image	12.7°
	Wind-erosion trace on soil Sol41 NaTeCam image	11.6°
Late-stage	Longitudinal dunes on the western horns of Bright Barchans in HiRIC and HiRISE images	290.4°-318.4° 301.4° in average
	Tip tail on the eastern horns of Bright Barchans in HiRIC and HiRISE images	282.6°-315.6° 300.8° in average
	Longitudinal dunes on Dune 2	278.7°-295.9° 283.9° in average
	Longitudinal dunes on Dune 5	282.4°-294.7° 290.4° in average
	Linear ridge on soil in Sol41 NaTeCam image	295.8° (Fig. 2o)

Note: Frequency distributions of wind direction showed the predominant wind direction of bright barchan-like dunes (Extended Data Figure 1c), indicating that the most concentrated wind direction is 12.5°. Since bright barchan-like dunes were modified by NW wind in the late-stage, the predominant wind direction measured from their morphology would shift. Therefore, the histogram of wind direction is negatively biased. The soil clasts are linearly arranged along the wind erosion traces formed in early-stage, and these wind erosion traces are less influenced by the late-stage wind field. The direction of the wind erosion traces measured from NaTeCam (Fig. 2o) is consistent with the most concentrated predominant wind direction of bright barchans. Therefore, 12.5° can be considered as the predominant wind direction of bright barchan-like dunes within the Zhurong landing site.

Supplementary Table 2 | The morphometric parameters of dunes 1-5

Dune number	Martian solar day	Crestline length(m)	Downwind length (m)	Maximum height (m)	Area (m^2)	Windward side (North slope)	Leeward side (South slope)	Indication of wind direction
1	Sol45, 46	48.9	8.0	0.9	250	11.9	13.9	14.0
2	Sol63, 64	61.8	8.9	/	351	/	/	354.0
3	Sol90, 91	29.0	5.5	0.5	131	7.9	9.5	3.0
4	Sol95, 99	66.1	11.0	1.0	435	8.8	13.9	11.0
5	Sol294, 297	45.9	11.7		404	/	/	9.3

Note: The maximum height, slope of the windward side and leeward side of dunes were measured from a 3-dimensional model derived from NaTeCam stereo images. Other parameters were measured from HiRIC images.

Supplementary Table 3 | Chemical composition of major elements for bright and dark sands of Dune 1

Sol	Targets [1]	Type	CaO	MgO	Al_2O_3	SiO_2	Fe_2O_3T	Na_2O	K_2O	TiO_2	Sum
47	D1-1	bright sand	2.87 ±0.78	7.46 ±2.46	4.58 ±1.16	52.04 ±2.32	15.59 ±0.74	1.01 ±0.28	0.43 ±0.59	1.80 ±0.38	85.78 ±3.80
47	D1-2	dark sand	3.26 ±0.56	5.85 ±1.81	6.44 ±1.12	55.91 ±2.21	15.49 ±1.20	1.31 ±0.22	0.69 ±0.56	1.64 ±0.34	90.59 ±3.41
Average Martian Soil [2]			6.37	8.32	9.71	45.41	18.59	2.73	0.44	0.90	92.47
Root mean squared error (RMSE, wt%) for the results of seven silicate pellets [3]			3.84	4.49	2.40	5.72	4.67	2.28	0.62	0.37	-

Note: Compositions are in wt% and 1σ uncertainty is used. (1) The target positions of MarSCoDe were marked in Extended Data Fig. 5a. (2) Average Martian Soil is a derived composition from soils analyzed in Viking and Pathfinder missions[87]; (3) RMSE was calculated from the deviation between the predicted value of the NGBoost model and the ground measured value for the major element content of seven silicate pellets, which were onboard calibration targets and included basalt, andesite, K-feldspar, hypersthene, olivine, montmorillonite and nontronite.

Supplementary Table 4 | The dating information related to bright barchans

Area	Hirise ID	Latitude (degree)	Areal extent （km^2）	Dune number	Crater number	Age (Myr)
N2	ESP_065997_2080	27.5	~19.2	26	0	-
N1	ESP_064533_2065	26.3	~14.1	54	1	-
Zhurong	ESP_069665_2055	25.1	~41.3	2262	38	~0.4-2
S1	ESP_046995_2045	24.0	~18.1	1027	8	~1-3
S2	ESP_062107_2035	23.5	~7.4	388	2	-

Supplementary Table 5 | The size and location of craters used for dune dating within landing region

Crater Number	Crater Diameter (m)	Longitude	Latitude
1	2.8	109.89	25.08
2	4.7	109.89	25.06
3	5.2	109.90	25.00
4	4.9	109.90	25.08
5	3.3	109.90	25.09
6	2.7	109.90	25.10
7	3.4	109.93	25.06
8	1.8	109.93	25.03
9	2.7	109.90	25.08
10	8.6	109.94	25.09
11	6.3	109.95	25.09
12	3.2	109.92	25.12
13	2.7	109.93	25.12
14	4.5	109.90	25.10
15	2.9	109.88	25.10
16	2.6	109.88	25.11
17	3.9	109.91	25.11
18	3.3	109.94	25.10
19	3.7	109.91	25.10
20	1.7	109.88	25.09
21	4.8	109.89	25.08
22	3.5	109.95	25.08
23	7.4	109.97	25.08
24	2.9	109.93	25.07
25	3.6	109.97	25.06
26	1.9	109.94	25.06
27	4.2	109.93	25.06
28	2.1	109.91	25.05
29	3.6	109.92	25.05
30	1.6	109.93	25.04
31	2.5	109.96	25.05
32	3.5	109.96	25.03
33	3.7	109.95	25.04
34	3.2	109.89	25.02
35	2.6	109.93	25.03
36	2.2	109.93	25.03
37	3.7	109.97	25.02
38	4.8	109.98	25.02

Supplementary Table 6 | The size and location of craters used for dune dating within S1 region

Crater Number	Crater Diameter (m)	Longitude	Latitude
a	3.6	109.10	24.03
b	4.0	109.05	24.03
c	4.0	109.07	24.02
d	3.2	109.12	24.05
e	7.1	109.08	24.05
f	7.1	109.05	24.06
g	5.6	109.11	24.07
h	4.5	109.04	24.06
